# The History and Capabilities of Herbal Simples

**Published:** 1890-12-27

**Authors:** W. T. Fernie


### The History and Capabilities of Herbal Simples.

By W. T. Fernie, M.D.

### XXV.—CRESS—*(continued)*.

The *Watercress*, Nasturtium officinale, is among cresses, to use an American simile, "the big dog with the brass collar," and "the finest toad in the puddle." This is because of its superlative medicinal worth, and its great popularity at table, with breakfast, or with the comfortable early evening meal, whereat, as Pope sang—

> "Thou, great Anna, whom three realms obey,
> Did sometimes counsel take, and sometimes tea."

Early writers called the plant shamrock, and common folk nowadays dub it the "'stertion." Zenophon advised the Persians to feed their children on watercresses, that they might grow in stature and have more active minds. Pliny said the watercress would make hair grow on bald places, and prevent it from falling out. The Latin name, *nasturtium*, has been given to the watercress through its pungency when smelt; from *nasus*, a nose, *tortus*, turned away, being as much as to say, "an herb that wriths, or twists the nose." For the same reason it is called *nasitord* in France. When bruised its leaves affect the eyes and nose almost like mustard. According to an analysis made recently in the School of Pharmacy, at Paris, the watercress contains a sulpho-nitrogenous oil, iodine, iron, phosphates, potash, certain other earthy salts, a bitter extract, and water. Its volatile oil, which is rich in sulphur, is the sulphocyanide of allyl. Thus the welcome plant is so constituted as to be particularly curative of scrofulous affections, especially in the spring time, when the bodily humours are apt to become vitiated. Dr. King Chambers tells us, in his "Diet in Health and Disease": "I feel sure that the infertility, pallor, fetid breath and bad teeth which characterize some of our town populations are to a great extent due to their inability to get fresh antiscorbutic vegetables as articles of diet, therefore I regard the watercress seller as one of the saviours of her country." Culpepper said, pithily, long ago, "They that will live in health may eat watercress if they please, and if they won't I cannot help it." Like oysters, the watercress is only in season when there is an "r" in the month; but some of our leading druggists make for medicinal use at other times a liquid extract and a spirituous juice of the officinal nasturtium. These preparations are of marked service in scorbutic cases, where weakness exists without wasting—as the chief symptom—often with spongy gums and some skin eruption. They are most palatable when taken with a little lemon juice. The watercress loses some of its pungent flavour and of its salutary qualities when cultivated, therefore it is more appetising and useful when freshly gathered from natural streams. But these streams should be free from contamination by sewage matter, or any drainage which might convey the germs of fever or of some other blood poison; for, as Dr. Smee admonishes us, the watercress plant acts as a brush in impure running brooks to detain around the stalks and leaves any dirty disease-bringing flocculi. On account of its medicinal constituents, the herb has been deservedly extolled as a remedy for tubercular consumption of the lungs. Haller says, "We have seen patients in deep declines cured by living almost entirely on this plant;" and it forms the chief ingredient of the Sirop Antiscorbutique given by the French faculty most successfully in scrofula or in other allied diseases. Its active principles are at their best when the plant is in flower, and the amount of essential oil increases according to the quantity of sunlight which the leaves obtain, the proportion of iron being determined by the quality of the water, and the measure of phosphates by the supply of dressing afforded. The leaves remain green when grown in the shade, but become of a purple brown when exposed to the sun. The expressed juice, which contains the peculiar taste and pungency of the herb, may be taken in doses of from one to two fluid ounces at each of the three principal meals, and it should always be had fresh. When combined with the juice of Scurvy Grass and of Seville oranges, it makes the popular antiscorbutic medicine known as "spring juices." Watercresses squeezed and laid against warts were said by the Saxon leeches to work a certain cure for these excrescences. In France the watercress is dipped in oil and vinegar, to be eaten at table with chicken or a steak. The Englishman takes it at his morning or evening meal with bread and butter, or at dinner in a salad. The leaf of the water parsnip, or fool's cress, which is injurious if eaten, resembles that of the watercress, and grows near it not infrequently. But the leaves of true watercress never embrace the stem of the plant as the leaf stalks of unwholesome plants which are like it generally do. Herrick, the joyous poet of "dull Devonshire" and "bad days," fondly loved this and its kindred herbs. He piously and tunefully made them the subject of a quaint grace before meat:—

> Lord! I confess, too, when I dine
> The pulse is Thine;
> And all those other bits that be
> There placed by Thee.
> The worts, the purslane, and the mess
> Of watercress.

This plant, the watercress, is justly popular with those who drink freely overnight, for its power of dissipating the fumes of the liquor, and of clearing off lethargic inaptitude for work in the morning; also for dispelling the tremors, and the foul taste induced by any inordinate tobacco-smoking.

